# Can the computer replace the adult for storybook reading? A meta-analysis on the effects of multimedia stories as compared to sharing print stories with an adult

**DOI:** 10.3389/fpsyg.2014.01366

**Published:** 2014-12-03

**Authors:** Zsofia K. Takacs, Elise K. Swart, Adriana G. Bus

**Affiliations:** Learning Problems and Impairments, Institute of Education and Child Studies, Leiden UniversityLeiden, Netherlands

**Keywords:** electronic books, multimedia storybooks, adult-child book sharing, story comprehension, vocabulary, meta-analysis

## Abstract

The present meta-analysis challenges the notion that young children necessarily need adult scaffolding in order to understand a narrative story and learn words as long as they encounter optimally designed multimedia stories. Including 29 studies and 1272 children, multimedia stories were found more beneficial than encounters with traditional story materials that did not include the help of an adult for story comprehension (*g*+ = 0.40, *k* = 18) as well as vocabulary (*g*+ = 0.30, *k* = 11). However, no significant differences were found between the learning outcomes of multimedia stories and sharing traditional print-like stories with an adult. It is concluded that multimedia features like animated illustrations, background music and sound effects provide similar scaffolding of story comprehension and word learning as an adult.

## Introduction

There is ample evidence that storybook reading is one of the most important sources of language and literacy development during the preschool, kindergarten and elementary school years (Bus et al., 1995; Mol and Bus, 2011). Adult guidance is a vital element of the traditional storybook reading paradigm. Beyond reading the print text, adults can involve the child in interactions regarding the story such as evoking comments from the child and providing feedback to their responses (Whitehurst et al., 1988). Such dialogic reading practices are more facilitative for children's vocabulary development than simply reading the story (Mol et al., 2008, 2009). Adult scaffolding is especially important for children below the age of four in order to enable their active involvement to promote story comprehension or vocabulary (Whitehurst et al., 1988).

Since the appearance of electronic stories that include an oral narration, children can “read” picture storybooks by themselves. Electronic storybooks include multimedia features that may support story understanding (Bus et al., in press; Takacs et al., under review). In accordance with the multimedia theory of learning (Mayer, 2005), we found evidence for the hypothesis that, if nonverbal information like animated illustrations, sound, and music are congruent with the story text, such multimedia features may facilitate story comprehension and learning new vocabulary (Bus et al., in press; Takacs et al., under review). For instance, animated illustrations are more helpful in explaining difficult words like “fanning” or “appearing” than a book with still illustrations. Animated scenes showing how someone fans a fire or how little crocodiles crawl out of their egg may be much more informative about these verbs than static pictures (Smeets and Bus, 2014; Smeets et al., 2014). Similarly, music and sound effects might depict abstract expressions or emotions like “puzzled” or “heartbroken” and thus contribute to children's meaning making processes (Smeets et al., 2014). According to the dual coding theory (Paivio, 2007), the human mind processes verbal and nonverbal information in two separate but interconnected channels. When nonverbal multimedia elements are processed simultaneous to the oral narration they may facilitate comprehension of verbal information and the story line.

The question arises: Can multimedia elements be just as effective as an adult as a scaffold for learning from book reading? We focus on vocabulary and story comprehension as outcome measures as those are most affected by multimedia features. Although book reading is shown to have benefits for other aspects of children's literacy development such as phonemic awareness and alphabet knowledge (Bus et al., 1995; Mol and Bus, 2011), those skills do not seem to benefit from multimedia elements (Korat and Shamir, 2007; Segal-Drori et al., 2010; Homer et al., 2014) as also appeared in a previous meta-analysis (Takacs et al., under review).

Intuitively it is assumed that support of an adult during storybook reading is superior to the benefits of multimedia features. The present meta-analysis challenged the notion that young children need adult scaffolding in order to understand a narrative and learn words as long as the multimedia material is optimally designed. We compared the effects of multimedia books including supplemental nonverbal information to shared book reading of print books. As motion and zooming may direct children's attention to a detail of the illustration in a similar way as an adult pointing at the detail and providing comments or explanations, multimedia may be just as beneficial in supporting story and language comprehension as interaction with an adult explaining the meanings of the story and sophisticated words in the narration.

We found several studies that do not show differences between how much children in this age range (preschool, kindergarten and elementary school ages) understand and learn from multimedia stories that they “read” by themselves as compared to sessions in which an adult reads a story to them (de Jong and Bus, 2004; Korat and Shamir, 2007; Silverman, 2013; Homer et al., 2014). Based on these findings the benefits of multimedia features seem comparable to adult scaffolding. However, there are also erratic outcomes in the literature. Shamir et al. (2012) found a significant advantage of working with an animated story over reading a print book with an adult on learning new vocabulary. In contrast, a study by Segers et al. (2004) showed that in a sample of immigrant children a teacher reading a storybook to the class was more facilitative of word learning as compared to a computer story with animations that children encountered on their own. The authors speculated that this might be explained by the computer software which included minimal animations. In the same study similar results were found for native speaking children. This mixed set of findings warrants a quantitative research synthesis on this issue.

Interactive features in electronic storybooks like dictionaries, hotspots and questions have been proposed to scaffold children's learning (McKenna et al., 1999; Caplovitz, 2005). However, in a recent meta-analysis (Takacs et al., under review) we found that interactive features, regardless of whether relevant to the story or not, decreased the benefits of electronic stories. We assume that these additions may require young children to switch between story comprehension and other tasks like playing games or listening to word explanations which may cause cognitive overload (Bus et al., in press). There is evidence showing that multimedia stories without or including only a limited number of interactive features are more advantageous for literacy skills than highly interactive electronic ones, whereas electronic books with a lot of interactive features are more advantageous for engaging children and prompting physical interaction (Chiong et al., 2012). We therefore did not include in the current meta-analysis studies of electronic books that include interactive features alone.

We expected that multimedia stories with motion pictures, sound, and music, all congruent with the story text, provide scaffolding that is equal to the support an adult offers during more traditional story sharing activities. Accordingly, we expected the following outcomes from book reading on children's comprehension of the story and word learning:

1. An overall advantage of multimedia stories as compared to print stories without support from an adult,
2. No advantage of multimedia stories when those are compared to print stories with support from an adult.

## Methods

### Operational definitions

The goal of the present study was to compare children's comprehension and word learning from narrative stories including multimedia elements to more traditional presentations of print stories with and without the support of an adult. Thus, we selected studies comparing stories including multimedia features to stories that were verbally presented (like during parent-child storybook sharing), either accompanied by static illustrations or not. We considered any verbally told story including multimedia features like animated or video illustrations, sound and background music a multimedia story. This broad definition of multimedia stories allowed for inclusion of studies testing television programs in addition to studies focusing on digital storybooks.

To be included there had to be a comparison condition in the experiment in which the same or a similar story was presented in a way that resembled the more traditional circumstances of children listening to stories, that is, listening to someone either telling a story or reading one from a picture storybook. To meet this criterion a comparison condition was required with either only orally presented stories or an oral rendition of the print in addition to a print book-like presentation with static illustrations, either supported by an adult (e.g., Korat and Shamir, 2007) or not (e.g., Smeets and Bus, 2014). We included studies assessing the differences between stories presented through “television” and “radio” formats, that is, an audiovisual and an audio presentation (e.g., Beagles-Roos and Gat, 1983; Gibbons et al., 1986) and studies that compared children encountering multimedia storybooks on their own with an adult reading the story from a print picture storybook to the child. In so far adults were involved they were either instructed to keep their interaction with the children to a minimum (e.g., Critelli, 2011) or they were encouraged to interact with the child during the reading, imitating a natural interactive shared reading session (e.g., de Jong and Bus, 2004; Korat and Shamir, 2007; Homer et al., 2014). In comparison conditions without adult the computer “read” the story while static pictures appeared on screen (e.g., Smeets and Bus, 2014).

#### Search strategy

We searched the databases of PsychInfo, ERIC and Web of Science for journal articles, reports and book chapters with a detailed search string including different terminology for literacy outcomes, technology-enhanced narrative stories and young children (see Appendix A in Supplementary Material). Secondary search involved inspection of the reference lists of review articles and the included articles for other suitable studies in addition to checking handbooks on technology and children's literacy development (see Appendix B in Supplementary Material for the list). Furthermore, we searched for dissertations and theses reporting data that might be suitable for the present meta-analysis. Over 3000 reports were scanned based on the titles and the abstracts, from which almost 300 full-text studies were checked. Finally, 29 studies were found eligible. For an overview of the procedure and the number of reports scanned see Appendix C in Supplementary Material.

When we could not find a full text we contacted the authors. If we did not succeed, we contacted authors referencing the study for a copy. Four studies (two conference papers and two reports) did not enter the meta-analysis because we could not locate those (Hudson, 1982; Meringoff, 1982; George and Schaer, 1986; Montouri, 1986).

### Inclusion criteria

According to our operational definitions, intervention studies were included based on the following criteria:

1. Experimental or (quasi-)experimental design with a contrast between a multimedia story and a comparison condition.
2. The study included a condition in which an orally presented narration was combined with multimedia features such as animations, music, and sound effects.
3. The comparison condition included an orally presented narration with or without static illustrations, with or without the support of an adult.
4. Participants were preschool-, kindergarten- or elementary school-aged children.
5. The study included as outcome measures the child's vocabulary and/or story comprehension.

There were no restrictions regarding the publication status of the manuscripts or the participants' country of origin as long as the article was written in English.

### Exclusion criteria

We excluded non-experimental studies (e.g., Kendeou et al., 2008), studies with foreign language learning (e.g., Tsou et al., 2006), and no eligible comparison condition (e.g., Trushell et al., 2003). We disregarded multimedia interventions focusing on expository texts (e.g., Silverman and Hines, 2009), stories with sign language (e.g., Wang and Paul, 2011) or without oral narration (e.g., Doty et al., 2001). We also excluded studies without any outcome measures (e.g., Reissner, 1996), and studies presenting the same data as in a study already included (Korat et al., 2009), or data only for a group of children and adults together (Pratt and MacKenzie-Keaing, 1985). Moreover, we excluded studies utilizing the support of an adult in the multimedia story condition (e.g., Korat et al., 2013) in order to assess whether adult support in traditional story sharing activities is more beneficial than the scaffolding that multimedia elements provide. See Appendix A in Supplementary Material for a prisma diagram of the literature search.

### Coding

We coded the following information:

1. Bibliographic information (e.g., authors, year, and title of study, published or not, kind of publication and the country in which the study was conducted),
2. Characteristics of the sample (e.g., the number of participants and mean age),
3. The design of the study (a. experimental or quasi-experimental, and b. between- or within-subject design),
4. Multimedia (e.g., animation, music and sound effects) and interactive features (e.g., hotspots, questions, games),
5. Features of comparison condition (only oral text or oral text and static illustrations)
6. Whether there was an adult in the comparison condition supporting the story encounter by interacting with the child (simply reading the text of the story to the child thus did not suffice as adult support),
7. The number of repeated interactions with the stories,
8. Outcome measures [a. story comprehension (retelling of the story or comprehension questions), b. vocabulary (expressive or receptive vocabulary, and whether assessing book-based or general vocabulary].

For information that was not available in the reports of the studies regarding the details of the multimedia stories we looked the software up on the Internet, for example checking videos and demos on Youtube.com. When more information was needed, the authors of the study were contacted via e-mail, if possible.

As shown in Table 1, whenever results were reported separately for subgroups of children, based on age (e.g., Williamson and Silvern, 1983; Pezdek et al., 1984), disadvantage status (e.g., Segers et al., 2004), or ability level (e.g., Verhallen and Bus, 2009b), separate effect sizes were calculated for the separate subgroups. When studies included two or more suitable multimedia conditions (e.g., Verhallen et al., 2006; Smeets and Bus, 2014) all contrasts were calculated. This was accomplished by dividing the number of participants in the comparison group by the number of suitable multimedia story conditions, without adjusting the scores, in order not to include children twice or more in the analyses (for a similar procedure see Bakermans-Kranenburg et al., 2003; Mol et al., 2008). In case there were more comparison conditions in a study, the condition most similar to a traditional print book reading activity was chosen (e.g., the “adult reading” condition in Terrell and Daniloff, 1996 and the “text and accompanying illustrations” condition in Williamson and Silvern, 1983).

**Table 1:** **Overview of the studies in the meta-analysis including the moderators and the effect sizes**.

**First author**	**Year**	**Age (years)**	**Technology-enhanced condition**	**Material**	**Relevant and irrelevant interactive features**	**Comparison condition**	**Illustrations**	**Interaction with an adult**	**Outcome measures**	**Average effect size (*g+*)**
Beagles-Roos	1983	6–10	“Television” (*n* = 24)	*A Story, A Story, Strega Nona* animated by Weston Woods Studios	No	“Radio” (*n* = 24)	No	No	Story comprehension (3 measures)	0.34
Critelli (thesis)	2011	4–6	“E-book” (*n* = 5)	*Bubbles* CD-ROM story	No	“Print book” (*n* = 5)	Yes	No	Story comprehension (1 measure)	0.00
de Jong	2002	4–6	“Computer book—restricted” (*n* = 12)	*P.B. Bear's Birthday Party* Bombilla	Yes, relevant and irrelevant	“Regular book” (*n* = 12)	Yes	No	Story comprehension (2 measures)	−0.27
de Jong	2004	4–6	“Electronic book” (*n* = 18)	*I'll Make You Well Again Said the Bear, Big Party for Tiger, Tiger and Bear in Traffic* Het Spectrum Electronic Publishing	Yes, relevant and irrelevant	“Printed book” (*n* = 18)	Yes	Yes	Story comprehension (1 measure)	−0.53
Gazella	2003	4–5	“Audiovisual” (*n* = 15)	Researcher-constructed story with videotaped puppets	No	“Audio-only” (*n* = 14)	No	No	Story comprehension (2 measures)	−0.11
Gibbons	1986	4–7	“Audiovisual” (*n* = 48)	Researcher-constructed stories with animated puppets	No	“Audio” (*n* = 48)	No	No	Story comprehension (1 measure)	0.52
Hayes	1986	3–6	“Television” (*n* = 22)	*How the Whale Got Its Throat*	No	“Radio” (*n* = 22)	No	No	Story comprehension (2 measures)	−0.45
				Television segment						
Homer	2014	5–7	“Kinect with activities” (*n* = 12)	*Children Make Terrible Pets* by Microsoft Games Studio for the Kinect	Yes, relevant and irrelevant	“Book reading” (*n* = 14)	Yes	Yes	Story comprehension (3 measures)	0.26
									Vocabulary–expressive (1 measure)	0.37
Korat	2007	5–6	“E-book” (*n* = 25)	*The tractor in the sandbox*	Yes, relevant	“Adult book reading” (*n* = 25)	Yes	Yes	Vocabulary–receptive (1 measure)	0.06
	Contr 1			Researcher-constructed						
				CD-ROM story						
Korat	2007	5–6	“E-book” (*n* = 25)	*The tractor in the sandbox*	Yes, relevant	“Adult book reading” (*n* = 25)	Yes	Yes	Vocabulary–receptive (1 measure)	−0.03
	Contr 2			Researcher-constructed						
				CD-ROM story						
Korat	2007	5–6	“E-book” (*n* = 50)	*The tractor in the sandbox*	Yes, relevant	“Adult book reading” (*n* = 50)	Yes	Yes	Story comprehension (1 measure)	−0.13
	Contr 1 and 2			Researcher-constructed						
				CD-ROM story						
Meringoff	1980	6–10	“Television” (*n* = 24)	*A Story, A Story* animated by Weston Woods Studios	No	“Book” (*n* = 24)	Yes	No	Story comprehension (3 measures)	−0.21
Neuman	1989	8 on average	“Televised version” (*n* = 17)	*Simon's Book*	No	“Storybook” (*n* = 10)	Yes	No	Story comprehension (3 measures)	0.32
Pezdek	1984A	8 on average	“Television” (*n* = 24)	*A Story, A Story, Strega Nona* animated by Weston Woods Studios	No	“Radio” (*n* = 24)	No	No	Story comprehension (3 measures)	1.02
	Contr 1									
Pezdek	1984A	11 on average	“Television” (*n* = 24)	*A Story, A Story, Strega Nona* animated by Weston Woods Studios	No	“Radio” (*n* = 24)	No	No	Story comprehension (3 measures)	0.55
	Contr 2									
Pezdek	1984B	5	“Audiovisual match” (*n* = 24)	Bert and Ernie and Big Birds segments from the TV show *Sesame Street*	No	“Audio only” (*n* = 24)	No	No	Story comprehension (2 measures)	0.59
Ricci	2002	6–7	“Interactive participant” (*n* = 16)	*The Ugly Duckling* Interactive story	Yes, relevant and irrelevant	“Audio only” (*n* = 17)	No	No	Story comprehension (4 measures)	0.23
Robb (dissertation)	2010	4–5	“Interactive reading alone” (*n* = 23)	*Curious George Goes to a Chocolate Factory* read with me DVD system	Yes, relevant	“Print book reading with parent” (*n* = 12)	Yes	Yes	Story comprehension (3 measures)	−0.06
	Contr 2									
Segers	2006	4–7	“Computer story” (*n* = 9)	*Treasure Chest with the Mouse*	Yes, relevant	“Teacher reading” (*n* = 8)	Yes	No	Vocabulary (2 measures)	0.62
				Researcher-constructed						
				CD-ROM stories						
Segers	2004	5 on average	“Computer reading” (*n* = 41)	*Treasure Chest with the Mouse*	Yes, relevant	“Teacher reading” (*n* = 41)	Yes	Yes	Story comprehension (1 measure)	−0.08
	Contr 1			Researcher-constructed						
				CD-ROM stories						
									Vocabulary—expressive (1 measure)	−0.02
Segers	2004	5 on average	“Computer reading” (*n* = 30)	*Treasure Chest with the Mouse*	Yes, relevant	“Teacher reading” (*n* = 30)	Yes	Yes	Story comprehension (1 measure)	−0.41
	Contr 2			Researcher-constructed						
				CD-ROM stories						
									Vocabulary—expressive (1 measure)	−0.18
Shamir	2012	5–7	“E-book” (*n* = 42)	*Confused Yuval*	Yes, relevant	“Printed book with adult” (*n* = 34)	Yes	Yes	Vocabulary—receptive (1 measures)	0.45
				Researcher-constructed						
				CD-ROM story						
Sharp	1995	5–6	“Helpful video” (*n* = 18)	Commercial video clips with researcher-constructed narratives	No	“No video” (*n* = 18)	No	No	Story comprehension (2 measures)	1.43
Silverman	2013	Kindergartners (5–6)	“Video” (*n* = 42)	*Arthur, Martha Speaks* TV shows	No	“Read aloud” (*n* = 36)	Yes	Yes	Vocabulary (2 measures)	−0.24
	Study 1									
Smeets	2014A	4–5	“Animated e-book” (*n* = 36)	*Pete on the Pavement, Bear Is In Love With Butterfly, Rokko the Crocodile, Bolder and the Boat, Cycling With Grandpa* Het Woeste Woud	No	“Static e-book” (*n* = 17)	Yes	No	Story comprehension (3 measures)	0.11
	Contr 1									
									Vocabulary (3 measures)	−0.18
Smeets	2014A	4–5	“Interactive animated e-book” (*n* = 33)	*Pete on the Pavement, Bear Is In Love With Butterfly, Rokko the Crocodile, Bolder and the Boat, Cycling With Grandpa* Het Woeste Woud	Yes, relevant	“Static e-book” (*n* = 16)	Yes	No	Story comprehension (3 measures)	0.19
	Contr 2									
									Vocabulary (3 measures)	0.13
Smeets	2014B	5–6	“Video book” (*n* = 28)	*Pete on the Pavement, Rokko the Crocodile, Bolder and the Boat, Little Kangaroo, Imitators, Dear Dear* Het Woeste Woud	No	“Static book” (*n* = 28)	Yes	No	Vocabulary—expressive (1 measure)	−0.25
	Experiment 1									
Smeets	2014B	5–7	“Video with music and sounds” (*n* = 21)	*Pete on the Pavement, Rokko the Crocodile, Bolder and the Boat, Little Kangaroo, Imitators, Dear Dear, Bear Is In Love With Butterfly, Sweat-Naughty Bear Baboen* Het Woeste Woud	No	“Static-no music or sound” (*n* = 21)	Yes	No	Vocabulary—expressive (1 measure)	0.07
	Experiment 2									
Terrell	1996	5	“Videotape” (*n* = 26)	An animated segment, including music, from a children's TV show with a researcher-constructed narrative	No	“Storybook” (*n* = 26)	Yes	No	Vocabulary (3 measures)	−0.58
Valkenburg	1997	6–10	“Television” (*n* = 64)	*Stega Nona Doctor de Soto* animated by Weston Woods Studios	No	“Radio” (*n* = 64)	No	No	Story comprehension (1 measure)	0.14
Verhallen	2006	5	“4× Multimedia” (*n* = 10)	*Winnie the Witch* Bombilla	No	“4× Static” (*n* = 10)	Yes	No	Story comprehension (1 measure)	1.16
	Contr 1									
									Vocabulary—expressive (1 measure)	1.07
Verhallen	2006	5	“1× Multimedia” (*n* = 10)	*Winnie the Witch* Bombilla	No	“1× Static” (*n* = 10)	Yes	No	Story comprehension (1 measure)	0.36
	Contr 2									
									Vocabulary—expressive (1 measure)	0.62
Verhallen	2009a	5 on average	“4× video” (*n* = 22)	*Winnie the Witch* Bombilla	No	“4× static” (*n* = 20)	Yes	No	Story comprehension (1 measure)	0.54
	Contr 1									
									Vocabulary—expressive (1 measure)	0.54
Verhallen and Bus	2009a	5 on average	“1× video” (*n* = 21)	*Winnie the Witch* Bombilla	No	“1× static” (*n* = 23)	Yes	No	Story comprehension (1 measure)	0.39
	Contr 2									
									Vocabulary—expressive (1 measure)	0.59
Verhallen (dissertation)	2009b	5	“Video” (*n* = 22)	*Winnie the Witch* Bombilla	No	“Static” (*n* = 20)	Yes	No	Story comprehension (2 measures)	0.98
	Contr 1									
									Vocabulary—expressive (1 measure)	0.02
Verhallen (dissertation)	2009b	5	“Video” (*n* = 13)	*Winnie the Witch* Bombilla	No	“Static” (*n* = 12)	Yes	No	Story comprehension (2 measures)	−0.23
	Contr 2									
									Vocabulary—expressive (1 measure)	0.89
Verhallen	2010	5	“Video” (*n* = 34)	*Winnie the Witch* Bombilla	No	“Static” (*n* = 29)	Yes	No	Vocabulary (2 measures)	0.36
Williamson	1983	Kindergartners (5–6)	“Animated film” (*n* = 10)	*Petunia* animated film of the story while researcher reads it	No	“Tradebook” (*n* = 10)	Yes	No	Story comprehension (2 measures)	0.15
	Contr 1									
Williamson	1983	Grade 3 (8–9)	“Animated film” (*n* = 10)	*Petunia* animated film of the story while researcher reads it	No	“Tradebook” (*n* = 10)	Yes	No	Story comprehension (2 measures)	1.00
	Contr 2									

In some cases (e.g., de Jong and Bus, 2002) one multimedia condition was chosen in order to have no less than 10 children in each condition in each contrast. In these cases we chose the most technology-enhanced condition (e.g., the “video with music and sound condition” in Experiment 2 in Smeets et al., 2014; the “Kinect with activities” condition in Homer et al., 2014; the “interactive” condition in Ricci and Beal, 2002; and the helpful video condition in Sharp et al., 1995). However, in the study by de Jong and Bus (2002) the “restricted/no-game electronic book” condition was chosen because when children had the option to play with the games, they hardly spent time listening to the story.

All studies were coded by two independent coders to assess inter-rater reliability. Agreement was on average κ = 0.80 (*SD* = 0.19).

### Meta-analytic procedures

Since different outcome measures were included with different scales, the standardized mean difference, Hedges' *g* was calculated for each contrast between the multimedia and comparison conditions. To calculate Hedges' *g* raw post-test means and standard deviations were favored over other statistics but in some cases only gain scores (e.g., Critelli, 2011) or only frequency distributions, *F, t* or chi-square statistics (e.g., Segers et al., 2006) were available. We entered the available statistics in the Comprehensive Meta-Analysis software (version 2.0; Borenstein et al., 2005) to calculate Hedges' *g* for each contrast for each outcome variable, as presented in Table 1. We preferred Hedges'*g* to alternatives because sample sizes were rather small (Lipsey and Wilson, 2001). If two or more vocabulary or story comprehension outcome measures were available in one study, the effect sizes for the different measures were averaged to compute an overall effect for each study. Interpretation of Hedge's *g* statistics is similar to that of Cohen's *d*. In previous meta-analyses of print exposure, effect sizes averaged around *d* = 0.50 (Bus et al., 1995; Mol and Bus, 2011). We expected an advantage of multimedia stories but lower than overall effects of print exposure. A positive effect size shows an advantage for the multimedia story condition, while a negative effect size suggests an advantage for the comparison condition.

The effect sizes for all vocabulary and story comprehension measures were inspected for outliers, which resulted in two outlying values (a *z*-score exceeding ± 3.29) (Tabachnick and Fidell, 2007). The two outliers were winsorized into a value of 0.01 higher, or lower in the case of the one negative effect size, than the highest or the lowest non-outlying effect size. Average effect sizes were computed over both outcome measures (story comprehension and vocabulary) and separately as well. This was decided because story comprehension and vocabulary measures are highly related constructs (Verhallen and Bus, 2009b; Smeets and Bus, 2014) since both tap on children's understanding and internalization of the narrative. At the same time, we intended to test any differences due to measurement issues so we also inspected average effect sizes separately for the different measures.

Overall effect sizes and 95% confidence intervals were computed based on the random effects model. This model was chosen because it is most conservative in handling between-study variability as a result of differences among study designs and intervention, and heterogeneity of the effect sizes (Lipsey and Wilson, 2001; Raudenbush, 2009). Heterogeneity of the effect sizes was estimated using the *Q*-statistic, with a significant *Q* indicating a heterogeneous effect, which means that more variability is found within the included studies than may be expected from sampling error on a subject level only (Lipsey and Wilson, 2001). Studies were weighted by the inverse of their variance, so that studies with larger sample sizes and more accurate estimates of population parameters had a greater weight on the mean effect size (Lipsey and Wilson, 2001; Shadish and Haddock, 2009).

It is referred to as publication bias when studies with significant and/or large findings are overrepresented because these are more likely to get published (Lipsey and Wilson, 2001; Borenstein et al., 2009). Publication bias can be observed by visual examination of the funnel plot. In case of asymmetry around the mean effect size, Duval and Tweedie's “Trim and Fill” procedure is widely used to adjust the overall effect size for publication bias (Duval and Tweedie, 2000).

Moderator analyses were performed, using a random effects model, to contrast subsamples based on different categorical study variables. Moderator analysis was only carried out when outcomes were heterogeneous according to *Q*-statistics. Only moderator variables were used that had at least four contrasts in one cell (cf. Bakermans-Kranenburg et al., 2003). For continuous study variables, as for example publication year, a meta-regression analysis was performed. Moderators were significant in cases of categorical variables, if *Q_between_*, or, for continuous variables, the regression model was significant.

## Results

### Descriptive statistics

A set of 29 studies including 38 contrasts, was eligible for this meta-analysis. It included 25 journal articles and four dissertations, all published between 1980 and 2014. All studies had an experimental design. A total of 1272 preschool and primary school children, aged 3–11 years, were included. The mean sample size in the primary studies was 41.03 children (*SD* = 20.00). The average number of repeated readings of the stories was 2.25 (*SD* = 1.63). Six of the studies only focused on vocabulary learning, 16 studies only included story comprehension measures and in seven studies both vocabulary and story comprehension were measured. From the 14 studies that included an adult in the comparison condition, one study (Robb, 2010) focused on parents, two on teachers (Segers et al., 2004, 2006), and in 11 studies the researchers themselves carried out the intervention. Thus, due to the low number of studies we were unable to test this variable as a moderator, which requires a minimum of four contrasts in each cell.

### Overall effect of technology in stories

For all included contrasts (see Table 1), an effect size of *g*+ = 0.19 was found, which represents a small but significant effect [*k* = 38; *SE* = 0.07; 95% CI = (0.06, 0.33); *p* < 0.01]. This effect was heterogeneous, *Q* (37) = 76.23, *p* < 0.01. After transforming the effect sizes into Fisher's *Z*, the funnel plot of the standard errors showed a symmetrical distribution around the overall effect and no studies had to be imputed using Duval and Tweedie's trim and fill procedure. Publication status (journal article vs. dissertation) was not a significant moderator (*Q_between_* = 0.05; *p* = 0.83), indicating the absence of any publication bias. To test for other biases, moderator analyses were performed for country and subject design (within vs. between) and meta-regression analyses were performed for publication date, number of repeated readings, sample size, and whether the children were from the preschool and kindergarten or the primary school age range. No significant regression models or moderators were found, indicating the absence of any bias.

Thirteen contrasts assessing story comprehension were based on measures of children's retelling of the story, 9 used questions and 8 utilized a mix of the two measures. For story comprehension, a significant effect of *g*+ = 0.23 was found when comparing multimedia stories to traditional story reading [*k* = 30; *SE* = 0.08; 95% CI = (0.07, 0.40); *p* < 0.01]. This effect was heterogeneous, *Q* (29) = 62.64, *p* < 0.001.

All contrasts assessing vocabulary focused on book-based word knowledge except for three that included a mix of measures regarding general and book-based vocabulary (Segers et al., 2006; Smeets and Bus, 2014). Furthermore, from the 20 vocabulary contrasts 12 assessed expressive word knowledge, 3 measured receptive knowledge and 5 contrasts used a mix of expressive and receptive vocabulary tests. For vocabulary learning, we found a marginally significant effect [*g*+ = 0.16; *k* = 20; *SE* = 0.09; 95% CI = (−0.02, 0.33); *p* = 0.08]. This effect was heterogeneous, *Q* (19) = 34.05, *p* < 0.02.

### Multimedia vs. adult support

**Figure 1:**
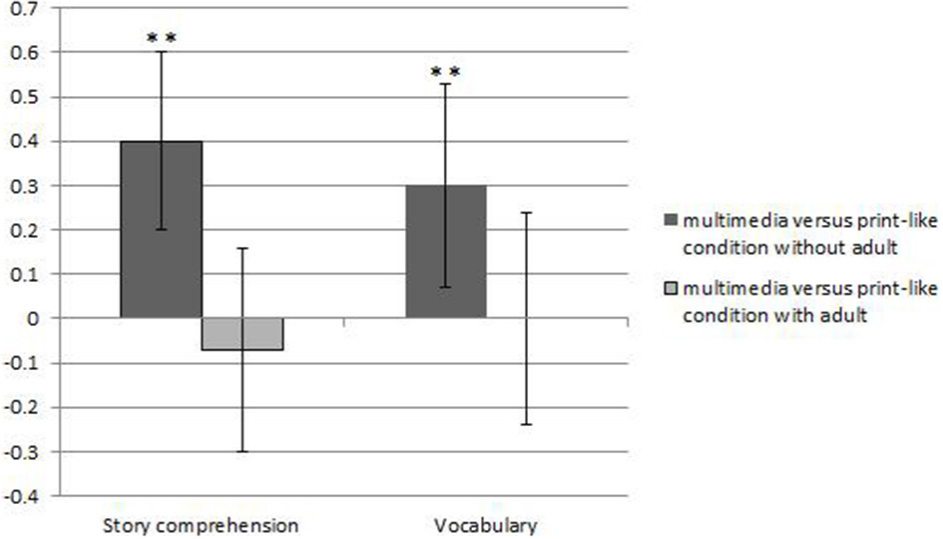
**The effect of multimedia added to stories as compared to a more traditional story sharing comparison condition with and without the support of an adult on story comprehension and vocabulary measures**. ^**^p ≤ 0.01.

To test whether multimedia can make up for the support of an adult we contrasted the multimedia condition with two types of comparison conditions: with and without the support of an adult. The presence of an adult in the print-like comparison condition was a significant moderator of the effect sizes, *Q_between_* (1) = 8.09, *p* < 0.01 (see Figure 1). Studies (*k* = 17) that compared multimedia stories to a print-like condition having an adult present to support the child showed no overall effect (*g*+ = −0.02; see Table 2). In contrast, studies (*k* = 21) that compared a multimedia story to a print-like condition in which no adult was present to support the child showed a significant moderate effect favoring the multimedia story (*g*+ = 0.35; see Table 2). A test for homogeneity indicated that the effect was heterogeneous, *Q* (20) = 39.92, *p* < 0.01.

**Table 2:** **Overview of the effects of multimedia stories as compared to children encountering traditional stories alone and with the support of an adult on the different outcome measures**.

**Outcome measure**	**Adult support in print-like condition**	**Number of contrasts included**	**Effect size (*g*+)**	**Standard error**	**95% confidence interval**	***p***	**Difference between the contrasts with a comparison condition including an adult or not (*Q_between_*)**
Overall	Yes	17	−0.02	0.10	(−0.22, 0.17)	0.81	*Q_between_* (1) = 8.09, *p* < 0.01
	No	21	0.35	0.08	(0.18, 0.51)	<0.01	
Story comprehension	Yes	12	−0.07	0.12	(−0.30, 0.16)	0.56	*Q_between_* (1) = 10.04; *p* < 0.01
	No	18	0.40	0.09	(0.22, 0.58)	<0.01	
Vocabulary	Yes	9	0.00	0.12	(−0.24, 0.24)	0.99	*Q_between_* (1) = 3.06; *p* = 0.08
	No	11	0.30	0.12	(0.07, 0.53)	0.01	

Only in two of the studies comparing multimedia stories with a traditional story condition without support of an adult the electronic book included both multimedia elements and interactive features (Ricci and Beal, 2002; Smeets and Bus, 2014). These two studies showed a non-significant effect of *g*+ = 0.19 [*SE* = 0.31; 95% CI = (−0.42, 0.80); *p* = 0.54]. The other 19 multimedia stories without interactive features showed a significant effect [*g*+ = 0.36; *SE* = 0.10; 95% CI = (0.18, 0.55); *p* < 0.01]. However, because of the low number of studies including interactivity in addition to multimedia features, no moderator analysis could be performed.

When we inspected results separately for story comprehension (*k* = 30) and vocabulary learning (*k* = 20), the presence of adult support in the print-like condition appeared to be a significant moderator for the effect of multimedia on story comprehension [*Q_between_* (1) = 10.04; *p* < 0.01], while for vocabulary this moderator was marginally significant [*Q_between_* (1) = 3.06; *p* = 0.08]. As shown in Table 2, multimedia stories showed a significant additional benefit as compared to children encountering print-like stories without the support of an adult both on story comprehension and vocabulary outcomes. With adult support in the comparison condition effect sizes were low for comprehension and vocabulary.

Effects for the different outcome measures were further analyzed for the group of studies that included a comparison condition without an adult. Separate meta-analyses for receptive and expressive vocabulary learning did result in a significant additional effect for expressive vocabulary [*g*+ = 0.34; *k* = 11; *SE* = 0.13; 95% CI = (0.09, 0.58); *p* < 0.01], but not for receptive vocabulary learning [*g*+ = −0.03; *k* = 3; *SE* = 0.16; 95% CI = (−0.34, 0.29); *p* = 0.87]. However, only in three contrasts receptive vocabulary was measured. Separate meta-analyses for the different kind of story comprehension measures showed comparable effects for both comprehension questions [*g*+ = 0.43; *k* = 8; *SE* = 0.14; 95% CI = (0.16, 0.70); *p* < 0.01] as well as for story retelling [*g*+ = 0.33; *k* = 15; *SE* = 0.12; 95% CI = (0.11, 0.56); *p* < 0.01].

Finally, to make sure that the significant benefit of multimedia stories over more traditional stories without support of an adult was not due to the absence of visual information in the comparison condition we tested the presence of illustrations in the comparison condition as a moderator. It was not a significant moderator, *Q_between_* (1) = 0.43, *p* = 0.51. Ten contrasts without adult support included a comparison condition with only oral text, showing a significant additional effect for the multimedia condition of *g*+ = 0.40 [*SE* = 0.13; 95% CI = (0.15, 0.64); *p* < 0.01]. However, also when the print-like condition did include static illustrations a significant positive additional effect was found for the multimedia condition [*g*+ = 0.30; *k* = 11; *SE* = 0.13; 95% CI = (0.04, 0.56), *p* = 0.02].

## Discussion

The present meta-analysis synthesized the empirical research regarding the effects of multimedia stories on young children's comprehension and word learning as compared to the support an adult provides during traditional storybook reading. In contrast to the storybook reading paradigm (e.g., Whitehurst et al., 1988), our results show that storybook reading is not necessarily a social activity with an adult present to support story comprehension and word learning. Multimedia stories proved to be more beneficial than encounters with traditional story materials that did not include the help of an adult. We found moderate effects for both story comprehension (*g*+ = 0.40, *k* = 18) as well as vocabulary (*g*+ = 0.30, *k* = 11). This confirms the findings of a previous meta-analysis showing an advantage of multimedia-enhanced stories over print-like comparison stories on children's literacy development (Takacs et al., under review). However, we found a non-significant effect of multimedia stories when the comparison condition included adult scaffolding. These findings indicate that multimedia elements provide scaffolding of children's understanding and word learning that is comparable to adult scaffolding during storybook reading.

Results were similar for both story comprehension and vocabulary. Furthermore, similar effect sizes were found for story comprehension questions, story retellings and expressive vocabulary measures. The only exception was receptive word knowledge for which we found no effect of multimedia as compared to traditional materials that children encountered alone. Comprehension of a word (receptive knowledge) precedes the ability to use the word or reflect on the meaning of the word (expressive knowledge) and may require more superficial learning (Verhallen and Bus, 2010). Encounters with traditional story materials appears to suffice for receptive word learning and multimedia cannot add to this. In line with this suggestion, a previous meta-analysis (Mol et al., 2008) found a smaller additional benefit of dialogic reading on receptive than on expressive vocabulary measures.

In regards to interactive features added to the multimedia stories, only two studies tested the difference between an interactive-multimedia story and a traditional story that children encountered alone (Ricci and Beal, 2002; Smeets and Bus, 2014). These studies show no difference between interactive and traditional stories, while studies with purely multimedia stories show an advantage of multimedia elements over children encountering traditional story materials alone. This finding, although preliminary due to the low number of studies, might suggest that it is not the interactive but the multimedia features that provide similar scaffolding as an adult for children's literacy experiences. In fact, Chiong et al. (2012) showed that e-books with many built-in interactive features are less stimulating for parent-child literacy-related interaction and children's story comprehension as compared to reading print books.

### Limitations

In the present study it was not possible to test whether the quality of guidance affects learning. It would have been interesting, for instance, to test whether parental guidance has a different effect than support from a researcher interacting with the child according to a transcript. Because of the low number of studies utilizing parental support such a comparison could not be made. In contrast to a researcher, parents might connect the story to the child's own experiences and thus be more effective than less personalized guidance offered by the researcher (Jones, 1996).

## Conclusion

In the present research synthesis including 29 studies and 1272 young children we found evidence that multimedia stories are more beneficial for story comprehension and word learning as compared to children encountering traditional stories without the support of an adult. In fact, we found no difference between the benefits of multimedia elements embedded in stories and reading traditional story materials while interacting with an adult. This suggests that multimedia features like animated illustrations, background music and sound effects provide similar scaffolding of story comprehension and word learning as an adult.

It is important to note that most commercially available electronic books are not necessarily similar to the ones used in the primary studies. They most often include a large amount of interactive features like hotspots and games (de Jong and Bus, 2003; Guernsey et al., 2012), which we found to have detrimental effects on children's story comprehension (Takacs et al., under review). Thus, the present research synthesis shows the potentials of electronic stories for children's language and literacy development but we cannot generalize the results to the available electronic stories on the market.

The presence of an adult does not have advantages for story comprehension and vocabulary learning beyond multimedia books but may have for other outcomes of book sharing. Children's reading motivation and attitude might be more facilitated by reading print storybooks with an adult (Sonnenschein and Munsterman, 2002; Baker, 2003). Furthermore, the parent-child relationship and children's socio-emotional development may benefit from sharing and discussing stories together (Bus, 2001; Laible, 2004; Aram and Aviram, 2009). These aspects of storybook reading were not investigated in the present study. However, at least as far as children's language and literacy development is concerned, children seem to benefit just as much from multimedia stories as from adult scaffolding. Thus, when there is no adult available to support children's encounters with a story, well-designed multimedia stories are an effective way to scaffold children's learning.

### Conflict of interest statement

The authors declare that the research was conducted in the absence of any commercial or financial relationships that could be construed as a potential conflict of interest.

## References

[B1] Aram D. Aviram S. (2009). Mothers' storybook reading and kindergartners' socioemotional and literacy development. Read. Psychol. 30, 175–194 10.1080/02702710802275348

[B2] Baker L. (2003). The role of parents in motivating struggling readers. Read. Writ. Q. 19, 87–106 10.1080/10573560308207

[B3] Bakermans-Kranenburg M. J. van IJzendoorn M. H. Juffer F. (2003). Less is more: meta-analyses of sensitivity and attachment interventions in early childhood. Psychol. Bull. 129, 195–215. 10.1037/0033-2909.129.2.195 12696839

[B4] Beagles-Roos J. Gat I. (1983). Specific impact of radio and television on children's story comprehension. J. Educ. Psychol. 75, 128–137 10.1037/0022-0663.75.1.128

[B5] Borenstein M. Hedges L. V. Higgins J. P. T. Rothstein H. R. (2009). Introduction to Meta-Analysis. Chichester: Wiley.

[B6] Borenstein M. Hedges L. V. Higgins J. P. T. Rothstein H. R. (2005). Comprehensive Meta Analysis (Version 2) [Computer Software]. Englewood, NJ: Biostat.

[B8] Bus A. G. (2001). Joint caregiver-child storybook reading: a route to literacy development, in Handbook of Early Literacy Research, Vol. 1., eds Neuman S. B. Dickinson D. K. (New York, NY: Guilford Press), 179–191.

[B9] Bus A. G. Takacs Z. K. Kegel C. A. T. (in press). Affordances and limitations of electronic storybooks for young children's emerging literacy. Dev. Rev.

[B10] Bus A. G. van IJzendoorn M. Pellegrini A. (1995). Joint book reading makes for success in learning to read: a meta-analysis on intergenerational transmission of literacy. Rev. Educ. Res. 65, 1–21 10.3102/00346543065001001

[B11] Caplovitz A. G. (2005). The Effects of Using an Electronic Talking Book on the Emergent Literacy Skills of Preschool Children (Doctoral Dissertation). Retrieved from Proquest Dissertations and Theses Global (UMI: 3187831).

[B12] Chiong C. Ree J. Takeuchi L. Erickson I. (2012). Print Books vs e-books. Comparing Parent-Child Co-Reading on Print, Basic and Enhanced e-book Platforms. New York, NY: The Joan Ganz Cooney Center at Sesame Workshop.

[B13] Critelli K. (2011). The Effect of Multimedia e-books on the Acquisition of Early Literacy Skills (Master's Thesis). Retrieved from Proquest Dissertations and Theses Global (UMI: 1500577).

[B14] de Jong M. T. Bus A. G. (2002). Quality of book-reading matters for emergent readers: an experiment with the same book in a regular or electronic format. J. Educ. Psychol. 94, 145–155 10.1037/0022-0663.94.1.145

[B15] de Jong M. T. Bus A. G. (2003). How well suited are electronic books to supporting literacy? J. Early Child. Literacy 3, 147–164 10.1177/14687984030032002

[B16] de Jong M. T. Bus A. G. (2004). The efficacy of electronic books in fostering kindergarten children's emergent story understanding. Read. Res. Q. 39, 378–393 10.1598/RRQ.39.4.2

[B17] Doty D. E. Popplewell S. R. Byers G. O. (2001). Interactive CD-ROM storybooks and young readers' reading comprehension. J. Res. Comput. Educ. 33, 374–384 10.1080/08886504.2001.10782322

[B18] Duval S. Tweedie R. (2000). A nonparametric “trim and fill” method for accounting for publication bias in meta-analysis. J. Am. Stat. Assoc. 95, 89–98 10.1080/01621459.2000.10473905

[B19] Gazella J. Stockman I. J. (2003). Children's story retelling under different modality and task conditions: implications for standardizing language sampling procedures. Am. J. Speech-Lang. Pathol. 12, 61–72. 10.1044/1058-0360(2003/053) 12680814

[B20] George Y. Schaer B. (1986). An investigation of imposed-induced imagery methods on kindergarten children's recall of prose content, in Paper presented at the Annual Meeting of the Mid-South Educational Research Association (Memphis).

[B21] Gibbons J. Anderson D. R. Smith R. Field D. E. Fischer C. (1986). Young children's recall and reconstruction of audio and audiovisual narratives. Child Dev. 57, 1014–1023. 10.2307/1130375 3757597

[B22] Guernsey L. Levine M. Chiong C. Severns M. (2012). Pioneering Literacy in the Digital Wild West: Empowering Parents and Educators. New York, NY: The Joan Ganz Cooney Center at Sesame Workshop.

[B23] Hayes D. S. Kelly S. B. Mandel M. (1986). Media differences in children's story synopses: radio and television contrasted. J. Educ. Psychol. 78, 341–346 10.1037/0022-0663.78.5.341

[B24] Homer B. D. Kinzer C. K. Plass J. L. Letourneau S. M. Hoffman D. Bormley M. (2014). Moved to learn: the effects of interactivity in a Kinect-based literacy game for beginning readers. Comp. Educ. 74, 37–49 10.1016/j.compedu.2014.01.007

[B25] Hudson T. J. (1982). Pictures, Spoken Word and Electronic Print: a Combination of Presentation Modes in Educational Television.

[B26] Jones R. (1996). Emerging Patterns of Literacy. A Multidisciplinary Perspective. London: Routledge.

[B27] Kendeou P. Bohn-Gettler C. White M. J. van den Broek P. (2008). Children's inference generation across different media. J. Res. Read. 31, 259–272 10.1111/j.1467-9817.2008.00370.x

[B28] Korat O. Segal-Drori O. Klien P. (2009). Electronic and printed books with and without adult support as sustaining emergent literacy. J. Educ. Comput. Res. 41, 453–475 10.2190/EC.41.4.d

[B29] Korat O. Shamir A. (2007). Electronic books versus adult readers: effects on children's emergent literacy as a function of social class. J. Comput. Assist. Learn. 23, 248–259 10.1111/j.1365-2729.2006.00213.x

[B30] Korat O. Shamir A. Heibal S. (2013). Expanding the boundaries of shared book reading: E-books and printed books in parent–child reading as support for children's language. First Lang. 33, 504–523 10.1177/0142723713503148

[B31] Laible D. (2004). Mother-child discourse in two contexts: links with child temperament, attachment security, and socioemotional competence. Dev. Psychol. 40, 979–992. 10.1037/0012-1649.40.6.979 15535752

[B32] Lipsey M. W. Wilson D. B. (2001). Practical Meta-Analysis. California, CA: Sage.

[B33] Mayer R. E. (2005). Principles for reducing extraneous processing in multimedia learning: Coherence, signaling, redundancy, spatial contiguity, and temporal contiguity principles, in The Cambridge Handbook of Multimedia Learning, ed Mayer R. E. (New York, NY: Cambridge University Press), 183–200.

[B34] McKenna M. C. Reinking D. Labbo L. D. Kieffer R. D. (1999). The electronic transformation of literacy and its implications for the struggling reader. Read. Writ. Q. 15, 111–126 10.1080/105735699278233

[B35] Meringoff L. K. (1980). Influence of the medium on children's story apprehension. J. Educ. Psychol. 72, 240–249. 10.1037/0022-0663.72.2.240 7365103

[B36] Meringoff L. K. (1982). What pictures can and can't do for children's story understanding, in Paper Presented at the Annual Meeting of the American Educational Research Association (New York, NY).

[B37] Mol S. E. Bus A. G. (2011). To read or not to read: a meta-analysis of print exposure from infancy to early adulthood. Psychol. Bull. 137, 267–296. 10.1037/a0021890 21219054

[B38a] Mol S. E. Bus A. G. de Jong M. T. (2009). Interactive book reading in early education: a tool to stimulate print knowledge as well as oral language. Rev. Educ. Res. 79, 979–1007 10.3102/0034654309332561

[B38] Mol S. E. Bus A. G. De Jong M. T. Smeets D. J. H. (2008). Added value of parent-child dialogic book readings: a meta-analysis. Early Educ. Dev. 19, 7–26 10.1080/10409280701838603

[B39] Montouri N. (1986). Video Storytime vs. Reading Storytime.

[B40] Neuman S. B. (1989). The impact of different media on children's story comprehension. Read. Res. Instruct. 28, 38–47 10.1080/19388078909557985

[B41] Paivio A. (2007). Mind and its Evolution: a Dual Coding Theoretical Approach. Mahwah, NJ: Lawrence Erlbaum Associates Publishers.

[B44] Pezdek K. Lehrer A. Simon S. (1984). The relationship between reading and cognitive processing of television and radio. Child Dev. 55, 2072–2082 10.2307/1129780

[B45] Pezdek K. Stevens E. (1984). Children's memory for auditory and visual information on television. Dev. Psychol. 20, 212–218 10.1037/0012-1649.20.2.212

[B47] Pratt M. W. MacKenzie-Keaing S. (1985). Organizing stories: effects of development and task difficulty on referential cohesion in narrative. Dev. Psychol. 21, 350–356 10.1037/0012-1649.21.2.350

[B48] Raudenbush S. W. (2009). Analyzing effect sizes: random-effects models, in The Handbook of Research Synthesis and Meta-Analysis, 2nd Edn., eds Cooper H. Hedges L. V. Valentine J. C. (New York, NY: Sage), 295–316.

[B49] Reissner L. A. (1996). Increasing beginning readers' reading success without increasing direct instruction time by using books on tape, in Paper presented at the Annual National Rural Special Education Conference (Bellingham, WA).

[B50] Ricci C. M. Beal C. R. (2002). The effect of interactive media on children's story memory. J. Educ. Psychol. 94, 138–144 10.1037/0022-0663.94.1.138

[B51] Robb M. B. (2010). New Ways of Reading: the Impact of an Interactive Book on Young Children's Story Comprehension and Parent-Child Dialogic Reading Behaviors (Doctoral Dissertation). Retrieved from Proquest Dissertations and Theses Global (UMI: 3426163).

[B52] Segal-Drori O. Korat O. Shamir A. Klein P. S. (2010). Reading electronic and printed books with and without adult instruction: effects on emergent reading. Read. Writ. 23, 913–930 10.1007/s11145-009-9182-x

[B53] Segers E. Nooijen M. de Moor J. (2006). Computer vocabulary training in kindergarten children with special needs. Int. J. Rehabil. Res. 29, 343–345. 10.1097/MRR.0b013e328010f4e0 17106354

[B54] Segers E. Takke L. Verhoeven L. (2004). Teacher-mediated versus computer-mediated storybook reading to children in native and multicultural kindergarten classrooms. School Effect. School Improv. 15, 215–226 10.1076/sesi.15.2.215.30430

[B55] Shadish W. R. Haddock C. K. (2009). Combining estimates of effect sizes, in The Handbook of Research Synthesis and Meta-Analysis, 2nd Edn., eds Cooper H. Hedges L. V. Valentine J. C. (New York, NY: Sage), 257–278.

[B56] Shamir A. Korat O. Fellah R. (2012). Promoting vocabulary, phonological awareness and concept about print among children at risk for learning disability: can e-books help? Read. Writ. 25, 45–69 10.1007/s11145-010-9247-x

[B57] Sharp D. L. M. Bransford J. D. Goldman S. R. Risko V. J. Kinzer C. K. Vye N. J. (1995). Dynamic visual support for story comprehension and mental model building by young, at-risk children. Educ. Technol. Res. Dev. 43, 25–42 10.1007/BF02300489

[B58] Silverman R. (2013). Investigating video as a means to promote vocabulary for at-risk children. Contemp. Educ. Psychol. 38, 170–179 10.1016/j.cedpsych.2013.03.001

[B59] Silverman R. Hines S. (2009). The effects of multimedia-enhanced instruction on the vocabulary of English-language learners and non-English-language learners in pre-kindergarten through second grade. J. Educ. Psychol. 101, 305–314 10.1037/a0014217

[B60] Smeets D. J. H. Bus A. G. (2014). The interactive animated e-book as a word learning device for kindergartners. Appl. Psycholinguist. Adv. [Epub ahead of print]. 10.1017/S0142716413000556

[B61] Smeets D. J. H. van Dijken M. J. Bus A. G. (2014). Using electronic storybooks to support word learning in children with severe language impairments. J. Learn. Disabil. 47, 435–449. 10.1177/0022219412467069 23213051

[B62] Sonnenschein S. Munsterman K. (2002). The influence of home-based reading interactions on 5-year-olds' reading motivations and early literacy development. Early Child. Res. Q. 17, 318–337 10.1016/S0885-2006(02)00167-9

[B64] Tabachnick B. G. Fidell A. S. (2007). Using Multivariate Statistics. Boston, MA: Pearson Education.

[B66] Terrell S. L. Daniloff R. (1996). Children's word learning using three modes of instruction. Percept. Motor Skills 83, 779–787 10.2466/pms.1996.83.3.779

[B67] Trushell J. Maitland A. Burrell C. (2003). Pupils' recall of an interactive storybook on CD-ROM. J. Comp. Assist. Learn. 19, 80–89 10.1046/j.0266-4909.2002.00008.x

[B68] Tsou W. Wang W. Tzeng Y. (2006). Applying a storytelling website in foreign language learning. Comp. Educ. 47, 17–28 10.1016/j.compedu.2004.08.013

[B69] Valkenburg P. M. Beentjes J. W. J. (1997). Children's creative imagination in response to radio and television stories. J. Commun. 47, 21–38 10.1111/j.1460-2466.1997.tb02704.x

[B70] Verhallen M. J. A. Bus A. G. de Jong M. T. (2006). The promise of multimedia stories for kindergarten children at risk. J. Educ. Psychol. 98, 410–419 10.1037/0022-0663.98.2.410

[B71] Verhallen M. J. A. J. Bus A. G. (2009a). Video storybook reading as a remedy for vocabulary deficits: outcomes and processes. J. Educ. Res. Online 1, 172–196.

[B72] Verhallen M. J. A. J. Bus A. G. (2009b). Video Storybooks: a Worthwhile Investment for all Young L2 Learners? (Unpublished doctoral dissertation). Leiden University, Leiden, Netherlands.

[B73] Verhallen M. J. A. J. Bus A. G. (2010). Low-income immigrant pupils learning vocabulary through digital picture storybooks. J. Educ. Psychol. 102, 54–61 10.1037/a0017133

[B74] Wang Y. Paul P. V. (2011). Integrating technology and reading instruction with children who are deaf or hard of hearing: the effectiveness of the Cornerstones Project. Am. Ann. Deaf 156, 56–68. 10.1353/aad.2011.0014 21644450

[B76] Whitehurst G. J. Falco F. L. Lonigan C. J. Fischel J. E. DeBaryshe B. D. Valdez-Menchaca M. C. (1988). Accelerating language development through picture book reading. Dev. Psychol. 24, 552–559 10.1037/0012-1649.24.4.552

[B77] Williamson P. A. Silvern S. B. (1983). The effects of text elaboration on children's aural language comprehension. Educ. Psychol. 3, 5–14 10.1080/0144341830030102

