# Supplementary material for: Can the computer replace the adult for storybook reading? A meta-analysis on the effects of multimedia stories as compared to sharing print stories with an adult
Source: Front Psychol. 2014 Dec 3;5:1366. doi: 10.3389/fpsyg.2014.01366 (PMC4253734; doi:10.3389/fpsyg.2014.01366)

1. **Supplementary Material**

**Appendix A Search terms for electronic database search.**

(literacy OR “emergent literacy” OR “early literacy” OR reading OR “early reading” OR “beginning reading” OR language OR vocabulary OR “story comprehension” OR “story retelling” OR attention OR engagement OR attitude) AND (computer OR technology OR e-book OR eStorybook OR digital OR CD-ROM OR multimedia OR multimodal OR “talking book” OR “electronic book” OR “living book” OR “living storybook” OR “dynamic book” OR animated OR animation OR video OR software OR tablet OR iPad OR television) AND (storybook OR book OR “picture storybook” OR narrative OR narration OR story OR stories) AND (children or kindergartner or preschooler)

**Appendix B Reference list of hand-searched books.**

Bus, A. G. & Neuman, S. B. (2009). *Multimedia and Literacy Development: Improving Achievement for Young Learners.* New York: Taylor & Francis Group.

Harris, K. R, Graham, S., Urdan, T., Bus, A. G., Major, S. & Swanson, H. L. (2011). *APA Educational Psychology Handbook, Volume 3: Application to Learning and Teaching.* Washington: American Psychological Association.

Kamil, M. L., Mosenthal, P. B., Pearson, P. D. & Barr, R. (2000). *Handbook of Reading Research: Volume III.* Mahwah, New Jersey: Lawrence Erlbaum Associates.

Kinzer, C. & Verhoeven, L. T. (2007). *Interactive Literacy Education: Facilitating Literacy Environments through Technology. Mahwah, New Jersey: Lawrence Erlbaum Associates.*

Mayer, R. E. (2005). *The Cambridge Handbook of Multimedia Learning.* New York: Cambridge University Press.

Martin, A. & Madigan, H. (2006). *Digital Literacies for Learning.* London: Facet Publishing.

McKenna, M. C., Labbo, L. D., Kieffer, R. D. & Reinking, D. *International Handbook of Literacy and Technology: Volume II.* Mahwah, NJ: Erlbaum.

Neuman, S. B. & Dickinson, D. K. (2011). *Handbook of Early Literacy Research, Volume 3.* New York: The Guilford Press.

Olson, D. R. & Torrance, O. (2009). *The Cambridge Handbook of Literacy.* New York: Cambridge University Press.

Shamir, A. & Korat, O. (2013). *Technology as a Support for Literacy Achievements for Children at Risk.* Dordrecht, The Netherlands: Springer Science + Business Media.

Stone, C. A., Silliman, E. R., Ehren, B. J. & Apel, K. (2004). *Handbook of Language and Literacy: Development and Disorders.* New York: Guilford Press.

**Appendix C PRISMA diagram of the literature search.**


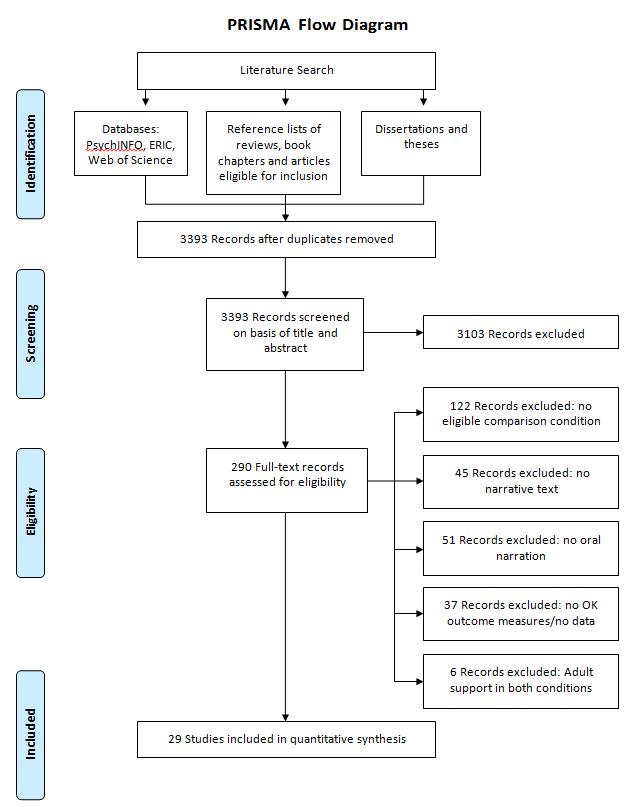

Supplement: Supplementary file 1 [file DataSheet1.DOCX]
